# Supplementary material for: Targeted Sub-Attomole Cancer Biomarker Detection Based on Phase Singularity 2D Nanomaterial-Enhanced Plasmonic Biosensor
Source: Nanomicro Lett. 2021 Mar 22;13:96. doi: 10.1007/s40820-021-00613-7 (PMC7985234; doi:10.1007/s40820-021-00613-7)
Supplement: Supplementary file 1 — Supplementary file1 (PDF 1324 KB) [file 40820_2021_613_MOESM1_ESM.pdf]

Supporting Information for

Targeted Sub-Attomole Cancer Biomarker Detection Based on Phase Singularity 2D Nanomaterial-Enhanced Plasmonic Biosensor

Yuye Wang[1,2], Shuwen Zeng[2,3*], Aurelian Crunteanu[2], Zhenming Xie[1], Georges Humbert[2], Libo Ma[4], Yuanyuan Wei[1], Aude Brunel[5], Barbara Bessette[5] , Jean-Christophe Orlianges[2], Fabrice Lalloué[5], Oliver G Schmidt[4], Nanfang Yu[3], Ho-Pui Ho[1*]

[1]Department of Biomedical Engineering, The Chinese University of Hong Kong, Shatin, New Territories, Hong Kong, People's Republic of China.

[2] CNRS, XLIM Research Institute, UMR 7252, University of Limoges, 123, Avenue Albert Thomas, Limoges, France

[3] Department of Applied Physics and Applied Mathematics, Columbia University, New York City, NY, USA

[4] Institute for Integrative Nanosciences, IFW Dresden, Helmholtzstr. 20, Dresden, Germany

[5] Faculty of Medicine, University of Limoges, EA3842-CAPTuR, GEIST, 2 rue du Dr Marcland , Limoges, France

* Corresponding authors. E-mail: aaron.ho@cuhk.edu.hk (Ho-Pui Ho), zeng@xlim.fr (Shuwen Zeng)

S1 Theoretical modeling

S1.1 Relationship between lateral position shift and reflectivity

In our simulation analysis, we calculated the reflection coefficient and lateral position shift under different incident angle using both Au-only substrate and atomically thin GST-on-Au substrate. To demonstrate the relationship between the reflectivity and lateral position shift more clearly, a plot showing their negative correlation was drawn through Fresnel equations and transfer matrix method (TMM) and calculated with a MATLAB programming (**Figure S1**). This explains more explicitly that the maximum lateral position shift is achieved at the minimum reflectivity. Therefore, it is very essential to enhance the zero-reflection effects of the sensing substrate.

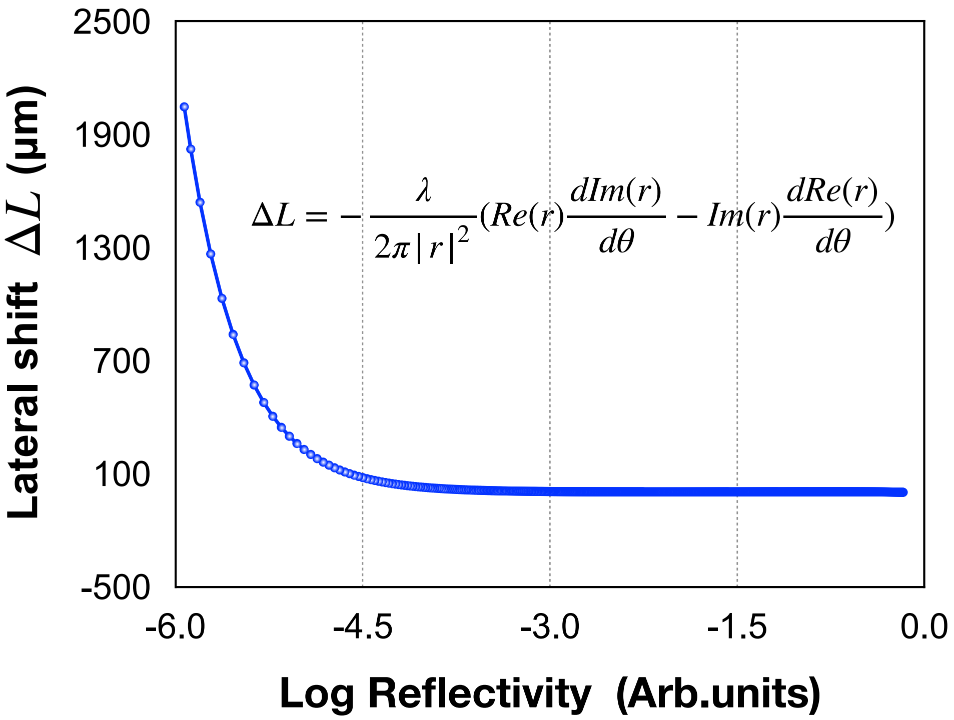

Figure S1. Relationship between plasmonic-based lateral position shift and reflectivity.

S1.2 Finite element analysis (FEA)

We use finite element analysis (FEA) (COMSOL Multiphysics 5.2) to study the electric field distribution on this 2D GST-on-Au sensing substrate at the resonance angle. As shown in **Figure S2** (a), a large electric field enhancement occurs at the sensing interface when surface plasmon resonance is excited. The resonance also results in a minimum reflectance and an enhanced lateral position shift in the reflected beam. We have conducted a comparison between the reflectivity and the lateral position shift of the Au-only substrate and our 2D GST-on-Au nanomaterial based substrate in **Figure S2** (b)(c). In both cases, the largest lateral position shift coincides with the minimum reflectivity point. An importance outcome of our analysis is that the addition of atomically thin GST material leads to a much deeper resonance dip. The corresponding maximum lateral position shift is 2107.33 μm, which is nearly 100 times larger than that associated with the case of using Au-only substrate. We can therefore assert that the atomically thin 2D GST layer will offer a superior sensitivity enhancement.

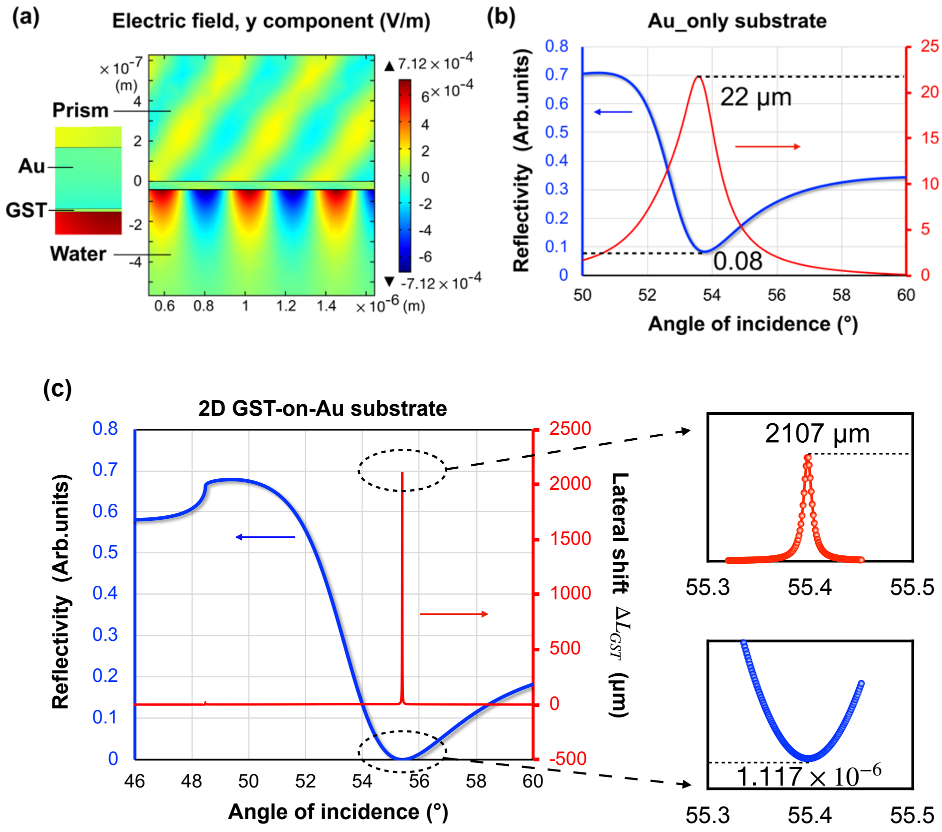

Figure S2. (a) Electric field distribution at SPR resonance angle 55.398◦. (b) Simulation results of reflectivity and lateral position shift of Au-only substrate. (c) Simulation results of reflectivity and lateral position shift of 2D GST-on-Au substrate with zoom-in figures on the right showing the largest lateral position shift and lowest reflectivity.

S2 Experimental

S2.1 Optical characterization

The dielectric constant of GST in relation to the photon energy was measured through spectroscopic ellipsometry. For our experimental configuration, which uses a He-Ne laser (632.8 nm), the dielectric constant was determined to be 13.00+11.10i.

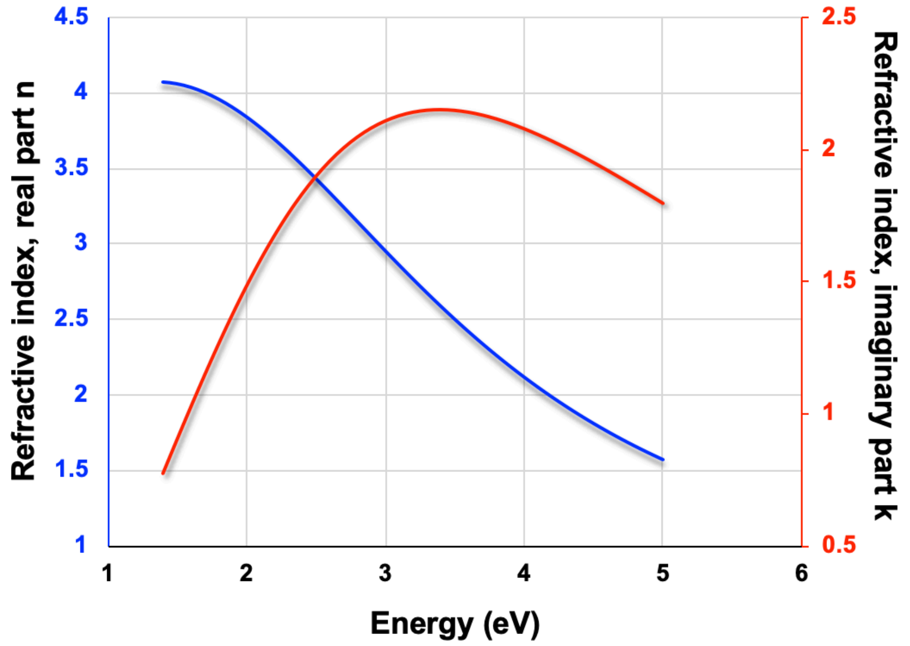

Figure S3. Refractive index measurement (real part and imaginary part) of GST under different photon energies.

S2.2 2D GST enhanced plasmonic sensing measurements

S2.2.1 Angular scanning reflectivity spectra in air

To evaluate the performance of the atomically thin GST-on-Au sensing substrate, we first measured its angular scanning reflectivity spectra in air. As a comparison, we also measured the Au-only substrate. The experimental results show good agreement with theoretical calculations, which confirms the reliability of our device and serves as a good calibration for the assessment of sensing performance. As shown in the **Figure S4**, the presence of GST material clearly leads to a deeper resonance dip (minimum intensity lowered by 50%).

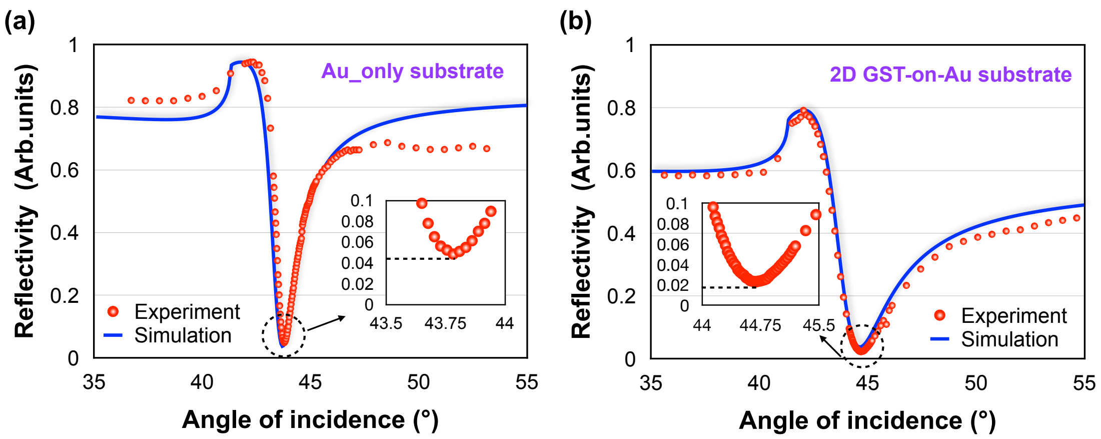

Figure S4. Reflectivity spectra measurement. (a) Au-only substrate (b) 2D GST-on-Au substrate.

S2.2.2 Standard sensor evaluation using glycerol solutions

In our experimental setup, the prism (SF11 glass, Edmund Optics) with a refractive index of 1.7786 was mounted on a high-precision rotation stage (PR01/M, Thorlabs) with the beam fixed at the surface plasmon resonance dip angle for achieving maximum lateral position shift. The microfluidic chamber containing sample solution was designed with the size of 10mm*10mm*0.5mm. The orientation of the detection screen (2D Lateral Effect Position Sensor, PDP90A, equipped with a K-Cube PSD Auto Aligner, KPA101, Thorlabs) is in a head-on direction towards the light path. The incident light beam from a He-Ne laser (632.8 nm, Newport) is split into *p*-polarized and *s*-polarized light beams through a polarized beam splitter (Thorlabs). As a standard sensor evaluation procedure, glycerol solutions of different concentration levels were injected into the microfluidic chamber. **Figure S5** shows the signals for both *s*-polarized light and *p*-polarized light acquired under different glycerol concentrations using atomically thin GST-on-Au substrate. As shown in the plot, the position of p-

polarized reflected light changes drastically while s-polarized reflected light remains in the same position upon injecting the glycerol/water solutions into the chamber. The signal is very stable due to the use of differential measurement scheme.

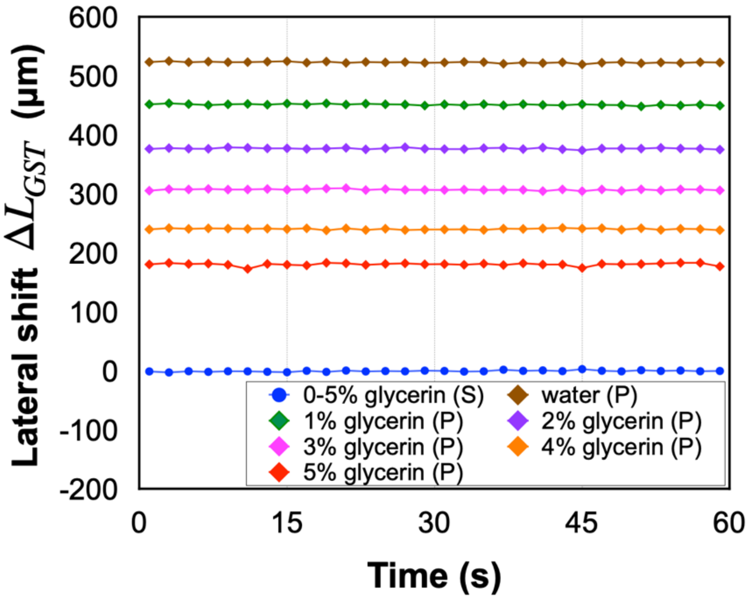

Figure S5. Lateral position shift of *p*-polarized and *s*-polarized light beam for different concentration levels of glycerol.

S2.2.3 Biomolecule (BSA) sensing performance

The real-time biosensing capability of our device is also demonstrated through monitoring binding of BSA biomolecules, which have relatively high molecular weight (66463 Da), at different concentration levels. Solutions of the biomolecules with different concentrations ranging from 10 fM to 10 μM were detected and recorded in **Figure S6** based on lateral position shift measurement, which shows a linear increase in lateral position shifts with increasing BSA

concentrations. Signal saturation will start when the concentration goes above 10^{-6} mol/L.

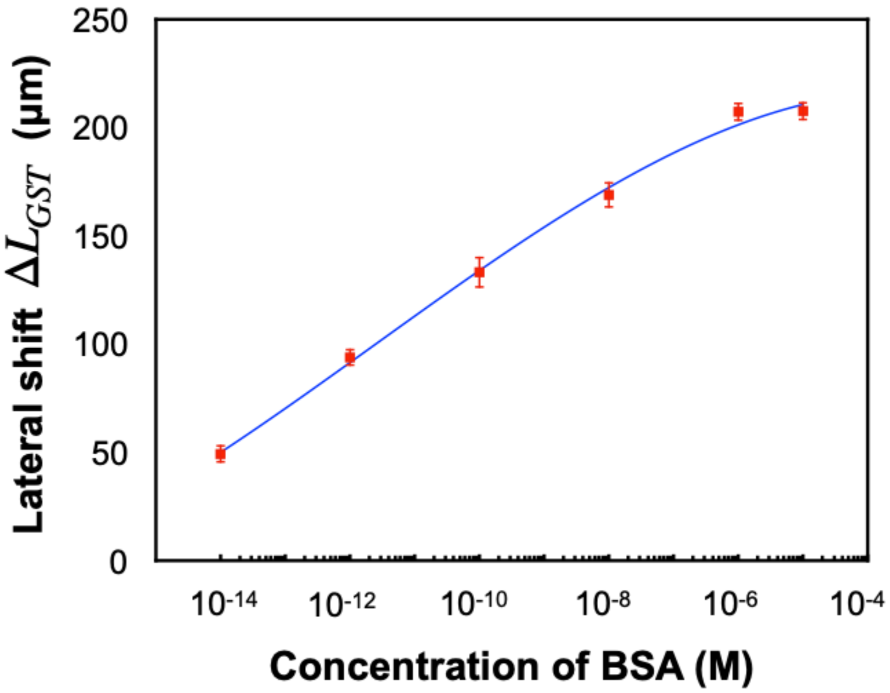

Figure S6. Detection of BSA molecules based on lateral position shift.

S2.2.4 Detection of non-specific binding

To demonstrate the specificity of our sensing device, the non-specific binding between TNF-alpha and BSA molecules has also been detected in comparison with the specific antibody-antigen binding. We carried out TNF-α (tumor necrosis factor α) antigen detection using a sandwich immunoassay strategy. After flowing antigen containing solutions to the sensing substrate coated with antibody solutions, we further injected the capture antibody - monoclonal anti-TNF antibody to the sensing substrate. The lateral position shift signal can be increased to 16.70 μm when flowing 10 pм antibodies. As a negative control experiment, we use BSA as the control antibody [1-4]. As shown in **Figure S7**, the lateral position shift signal change when flowing BSA

with a large concentration (10^5 times higher than anti-TNF antibody) is much smaller compared to flowing antibody, which shows the high specificity of our sensing device.

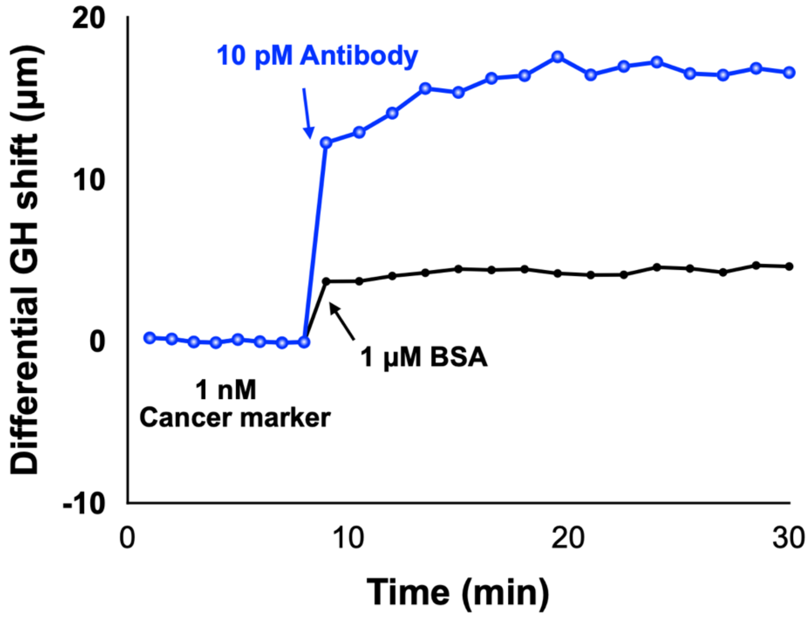

Figure S7. Detection of specific and non-specific binding based on lateral position shift. Blue curve shows the signal change when flowing capture antibody - monoclonal anti-TNF antibody to the sensing substrate while the black curve shows the signal change of flowing BSA as control antibody.

S2.2.5 Cancer marker detection

The differential lateral position shift signals acquired during TNF-α detection with replicate measurements were summarized. The lateral position shift signal can reach 6.52 μm for 1 fM cancer marker and 53.15 μm for 1 nM cancer marker using GST-on-Au substrate.

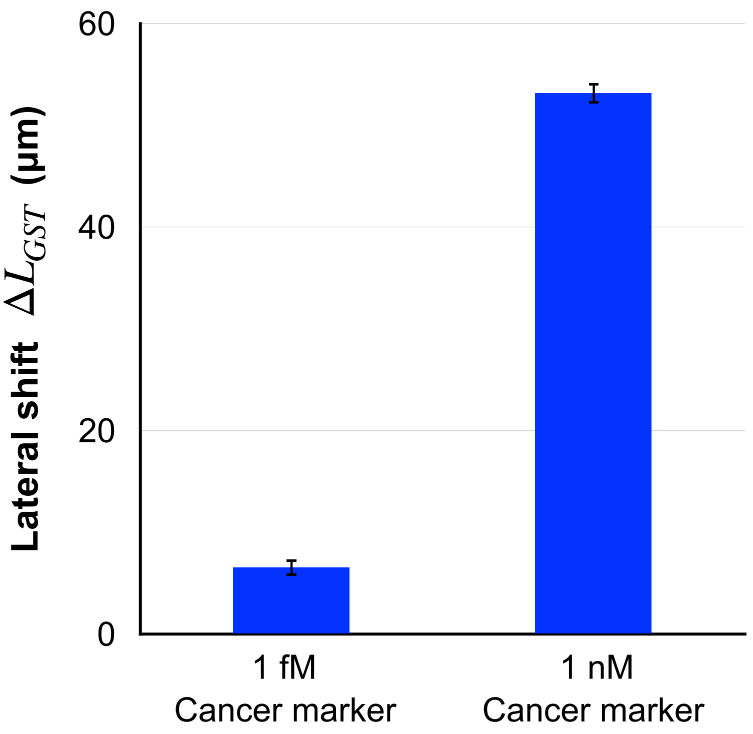

Figure S8. Detection of TNF-α based on lateral position shift.

Supplementary References

[S1] F. Yesilkoy, R. A. Terborg, J. Pello, A. A. Belushkin, Y. Jahani, V. Pruneri et al., Phase-sensitive plasmonic biosensor using a portable and large field-of-view interferometric microarray imager. Light Sci. Appl. 7, 17152 (2018). https://doi.org/10.1038/lsa.2017.152

[S2] I. Lee, X. Luo, X. T. Cui, M. Yun, Highly sensitive single polyaniline nanowire biosensor for the detection of immunoglobulin G and myoglobin. Biosens. Bioelectron. 26, 3297 (2011). https://doi.org/10.1016/j.bios.2011.01.001

[S3] R. Ohno, H. Ohnuki, H. Wang, T. Yokoyama, H. Endo et al., Electrochemical impedance spectroscopy biosensor with interdigitated electrode for detection of human immunoglobulin A. Biosens. Bioelectron. 40, 422 (2013). https://doi.org/10.1016/j.bios.2012.07.052

[S4] A. Bakhmachuk, O. Gorbatiuk, A. Rachkov, B. Dons'koi, R. Khristosenko et al.,

Surface plasmon resonance investigations of bioselective element based on the recombinant protein A for immunoglobulin detection. Nanoscale Res. Lett. 12, 112 (2017). https://doi.org/10.1186/s11671-017-1903-5
